# Supplementary material for: Building a framework for fake news detection in the health domain
Source: PLoS One. 2024 Jul 8;19(7):e0305362. doi: 10.1371/journal.pone.0305362 (PMC11230534; doi:10.1371/journal.pone.0305362)
Supplement: S1 Data — (DOCX) [file pone.0305362.s001.docx]

S1 Data The dataset, with sentence-level annotation information, is available at the link https://doi.org/10.5281/zenodo.10802196
