# Supplementary material for: Building a framework for fake news detection in the health domain
Source: PLoS One. 2024 Jul 8;19(7):e0305362. doi: 10.1371/journal.pone.0305362 (PMC11230534; doi:10.1371/journal.pone.0305362)
Supplement: S2 Data — The source code is available in the following repositories: Backend: https://github.com/jrmtnez/hnfc-agentFrontend: https://github.com/jrmtnez/hnfc-site (DOCX) [file pone.0305362.s002.docx]

S2 Data The source code is available in the following repositories:

• Backend: https://github.com/jrmtnez/hnfc-agent

• Frontend: https://github.com/jrmtnez/hnfc-site
