# Supplementary material for: Building a framework for fake news detection in the health domain
Source: PLoS One. 2024 Jul 8;19(7):e0305362. doi: 10.1371/journal.pone.0305362 (PMC11230534; doi:10.1371/journal.pone.0305362)
Supplement: Attachment — Submitted filename: response_to_reviewers.pdf [file pone.0305362.s003.pdf]

Authors' Response to the Reviewer's Comments

Article:: PONE-D-23-40818
Title: Building a Framework for Fake News Detection in the Health Domain
Authors: Juan R. Martinez-Rico, J. Martinez-Romo, and Lourdes Araujo

The authors thank the editor and reviewers for their comments which greatly helped them to improve the contents of the paper. We have prepared a revised version of the paper according to their suggestions. Changes and new parts appear in red in this version.

Next, we will show the comments/suggestions from the reviewer in italics and our response in regular typeface.

Responses to the comments of the editor

Thank you so much for your comments. We have followed the suggestions and indications of the editor and reviewers and have modified the article, mainly in the following aspects:

1. Please ensure that your manuscript meets PLOS ONE's style requirements, including those for file naming.

- We have revised the manuscript to fit the latex template the journal provides.

2. In your Methods section, please include additional information about your dataset and ensure that you have included a statement specifying whether the collection and analysis method complied with the terms and conditions for the source of the data.

- The data used in this work comprises articles gathered from various Internet sources, including magazine pages or other forums that may hold distribution rights. Thus, in line with the usual procedure in this context, when utilizing data accessible on the Internet that may be subject to distribution rights, we have made available a dataset composed of the URLs that allow access to the original news items, and sentence-level check-worthy and fact-checking annotations for the sentences in each of those articles. In this way, we avoid disseminating data that may be subject to copyright but at the same time we provide the annotation information that we have generated.

- The “Methods” section has also been modified, eliminating the old mention to the dataset in “Compiled dataset structure and statistics”:

~~*The dataset will be provided to those who request it via email of the authors, bearing in mind that it should only be used for research purposes and cannot be redistributed.*~~

And adding information about its public availability, as well as the URL from where it can be downloaded in “Data collection and annotation process”:

The methodology presented in this paper has allowed us to gather a collection of news items and manually annotate them, to compile an evaluation corpus with which to develop and analyze the performance of our systems. This section describes how we collected the news items, the annotation criteria used, and some statistical data from the corpus. The dataset is publicly available and the access link can be found in Section 8. It is composed of the URLs that allow access to the original news items, and for each of them, the sentence-level annotation of both: fact-checking and check-worthiness. Its structure and statistics are described later in Section 5.1.4.

3. Thank you for stating the following financial disclosure:

“INITIALS: LAS, JMR GRANT NUMBER: PID2019-106942RB-C32 FUNDER: Spanish Ministry of Science and Innovation URL FUNDER: https://www.ciencia.gob.es/

INITIALS: JMR, LAS GRANT NUMBER: TED2021-130398B-C21 FUNDER: Spanish Ministry of Science and Innovation URL FUNDER: https://www.ciencia.gob.es/

INITIALS: JMR, LAS GRANT NUMBER: PID2022-136522OB-C21 FUNDER: Spanish Ministry of Science and Innovation URL FUNDER: https://www.ciencia.gob.es/

INITIALS: LAS GRANT NUMBER: RAICES (IMIENS 2022) FUNDER: IMIENS (Instituto Mixto de Investigación-Escuela Nacional de Sanidad) URL FUNDER: https://www.imiens.es/”

Please state what role the funders took in the study. If the funders had no role, please state: “The funders had no role in study design, data collection and analysis, decision to publish, or preparation of the manuscript.”

If this statement is not correct you must amend it as needed.

Please include this amended Role of Funder statement in your cover letter; we will change the online submission form on your behalf.

- Thank you for pointing this out. The financial disclosure should look like this:

INITIALS: LAS, JMR
GRANT NUMBER: PID2019-106942RB-C32

FUNDER: Spanish Ministry of Science and Innovation
URL FUNDER: https://www.ciencia.gob.es/
The funder had no role in study design, data collection and analysis, decision to publish, or preparation of the manuscript.

INITIALS: JMR, LAS
GRANT NUMBER: TED2021-130398B-C21
FUNDER: Spanish Ministry of Science and Innovation
URL FUNDER: https://www.ciencia.gob.es/
The funder had no role in study design, data collection and analysis, decision to publish, or preparation of the manuscript.

INITIALS: JMR, LAS
GRANT NUMBER: PID2022-136522OB-C21
FUNDER: Spanish Ministry of Science and Innovation
URL FUNDER: https://www.ciencia.gob.es/
The funder had no role in study design, data collection and analysis, decision to publish, or preparation of the manuscript.

INITIALS: LAS
GRANT NUMBER: RAICES (IMIENS 2022)
FUNDER: IMIENS (Instituto Mixto de Investigación-Escuela Nacional de Sanidad)
URL FUNDER: https://www.imiens.es/
The funder had no role in study design, data collection and analysis, decision to publish, or preparation of the manuscript.

4. Thank you for stating in your Funding Statement:

"This work has been partially supported by the Spanish Ministry of Science and Innovation within the DOTT-HEALTH Project (MCI/AEI/FEDER, UE) under Grant PID2019-106942RB-C32, OBSER-MENH Project (MCIN/AEI/10.13039/501100011033 and NextGenerationEU"/PRTR) under Grant TED2021-130398B-C21, and EDHER-MED under grant PID2022-136522OB-C21 as well as project RAICES (IMIENS 2022)."

*Please provide an amended statement that declares *all* the funding or sources of support (whether external or internal to your organization) received during this study, as detailed online in our guide for authors at http://journals.plos.org/plosone/s/submit-now. Please also include the statement "There was no additional external funding received for this study." in your updated Funding Statement.*

Please include your amended Funding Statement within your cover letter. We will change the online submission form on your behalf.

- We have included the amended statement in the cover letter as requested.

5. Thank you for stating the following in the Acknowledgments Section of your manuscript:

"This work has been partially supported by the Spanish Ministry of Science and Innovation within the DOTT-HEALTH Project (MCI/AEI/FEDER, UE) under Grant PID2019-106942RB-C32, OBSER-MENH Project (MCIN/AEI/10.13039/501100011033 and NextGenerationEU"/PRTR) under Grant TED2021-130398B-C21, and EDHER-MED under grant PID2022-136522OB-C21 as well as project RAICES (IMIENS 2022)."

We note that you have provided funding information that is not currently declared in your Funding Statement. However, funding information should not appear in the Acknowledgments section or other areas of your manuscript. We will only publish funding information present in the Funding Statement section of the online submission form.

Please remove any funding-related text from the manuscript and let us know how you would like to update your Funding Statement. Currently, your Funding Statement reads as follows:

"INITIALS: LAS, JMR GRANT NUMBER: PID2019-106942RB-C32 FUNDER: Spanish Ministry of Science and Innovation URL FUNDER: https://www.ciencia.gob.es/

INITIALS: JMR, LAS GRANT NUMBER: TED2021-130398B-C21 FUNDER: Spanish Ministry of Science and Innovation URL FUNDER: https://www.ciencia.gob.es/

INITIALS: JMR, LAS GRANT NUMBER: PID2022-136522OB-C21 FUNDER: Spanish Ministry of Science and Innovation URL FUNDER: https://www.ciencia.gob.es/

INITIALS: LAS GRANT NUMBER: RAICES (IMIENS 2022) FUNDER: IMIENS (Instituto Mixto de Investigación-Escuela Nacional de Sanidad) URL FUNDER: https://www.imiens.es/"

Please include your amended statements within your cover letter; we will change the online submission form on your behalf.

- We have removed the funding information from the manuscript and we have included the amended statement in the cover letter as requested.

6. When completing the data availability statement of the submission form, you indicated that you will make your data available on acceptance. We strongly recommend all authors decide on a data sharing plan before acceptance, as the

process can be lengthy and hold up publication timelines. Please note that, though access restrictions are acceptable now, your entire data will need to be made freely accessible if your manuscript is accepted for publication. This policy applies to all data except where public deposition would breach compliance with the protocol approved by your research ethics board. If you are unable to adhere to our open data policy, please kindly revise your statement to explain your reasoning and we will seek the editor's input on an exemption. Please be assured that, once you have provided your new statement, the assessment of your exemption will not hold up the peer review process.

- We have made public the dataset with the URLs that allow access to the data used in the work, and the annotations at the sentence level. As stated above, the dataset has been collected from public websites using scrapping tools, as is usually done in this area of research focused on the detection of fake news and disinformation. For this reason, we cannot redistribute directly the content of these websites that may hold distribution rights. However, we have made public all the information necessary for the experiments to be replicated including both the code and the annotated data used.

7. We notice that your supplementary [figures/tables] are included in the manuscript file. Please remove them and upload them with the file type 'Supporting Information'. Please ensure that each Supporting Information file has a legend listed in the manuscript after the references list.

- We have removed the figures from the manuscript and referenced them as supporting information, creating the necessary tiff files using the PACE tool that you provide. Regarding the tables, after seeing the latex example template we understand that they can be in the manuscript as latex tables and it is not necessary to take them to external files. Also, some of the tables have citations so it is difficult to take them to an external file.

Responses to the additional comments of the editor

1. Is the manuscript technically sound, and do the data support the conclusions?

The manuscript must describe a technically sound piece of scientific research with data that supports the conclusions. Experiments must have been conducted

rigorously, with appropriate controls, replication, and sample sizes. The conclusions must be drawn appropriately based on the data presented.

Reviewer #1: Partly

Reviewer #2: Partly

- We have significantly improved the manuscript thanks to the suggestions indicated by the reviewers.

2. Has the statistical analysis been performed appropriately and rigorously?

Reviewer #1: Yes

Reviewer #2: No

- We have added standard deviation values to all measurements as requested.

3. Have the authors made all data underlying the findings in their manuscript fully available?

The PLOS Data policy requires authors to make all data underlying the findings described in their manuscript fully available without restriction, with rare exception (please refer to the Data Availability Statement in the manuscript PDF file). The data should be provided as part of the manuscript or its supporting information, or deposited to a public repository. For example, in addition to summary statistics, the data points behind means, medians and variance measures should be available. If there are restrictions on publicly sharing data—e.g. participant privacy or use of data from a third party—those must be specified.

Reviewer #1: No

Reviewer #2: Yes

- We have made both the source code and the dataset public, as requested.

4. Is the manuscript presented in an intelligible fashion and written in standard English?

PLOS ONE does not copyedit accepted manuscripts, so the language in submitted articles must be clear, correct, and unambiguous. Any typographical or grammatical errors should be corrected at revision, so please note any specific errors here.

Reviewer #1: Yes

Reviewer #2: Yes

- Thank you for the positive feedback.

Responses to the comments of reviewer 1

Reviewer #1: In this paper, the authors tackle the issue of fake news detection in the health domain by proposing a pipeline to automatically harvest health-related news and annotate them at the sentence level for Check-Worthiness (CW) and Fact-Checking (FC) in order to perform fake news detection. In order to train and evaluate their approach, a novel dataset is presented, namely KEANE, containing 327 articles belonging to the health-domain and collected from Snopes and Politifact, annotated both at the sentence and at the document level. Main contribution of this work is to train and evaluate on this corpus a fake news detection system that performs CW in order to filter relevant sentences and FC in order to spot sentences containing false claims. For CW, an uncased BERT model trained on the general domain proves to be the most efficient model. For FC, the best results are obtained with an ensemble model that combines three features: the text of the sentence, a subject-predicate-object triple extracted from the sentence, and a sequence of CUI identifiers extracted by mapping sentence tokens to the UMLS terminology. Despite being outperformed by transformers trained on the biomedical domain, this approach shows competitive performances even on unseen data. Moreover, the authors claim that this drawback is compensated by the fact that the proposed method offers more explainability since it identifies the specific sentences in a news article which support false claims.

Main strengths of this paper include a clear motivation on why sentence-level FC is relevant, solid grounding on the literature about fake news detection and the level of detail used to explain both the data collection process and the classification approach. Moreover, the methodology for the data collection is novel and the annotation practices are soundly justified and clearly explained. The approach, albeit its complexity, is presented in detail and reproducible, assuming that the authors provide the data and the documentation on the source code publicly. However, there are significant weaknesses in the manuscript. Sometimes the research is not presented in a scientifically thorough way. Significant details that justify certain methodological choices are lacking and the results, as they are presented, do not fully support the statements of the paper. Moreover, the authors claim that the data will not be released without restrictions, which is not compli-

ant with the PLOS Data Policy. My suggestion for the authors is to publish the data with an unrestricted license on a repository such as Zenodo, which provides a citable DOI and ensures long-term preservation. My final advice is that the article should be accepted under major revisions, upon the publication of the data and after addressing the following points of criticism.

- Thank you very much for your comments. In the following sections we hope to have resolved the problems you describe. Regarding the publication of the dataset, as we indicate in this same document, the content may be subject to publication restrictions by the sources. Therefore, we have made public the dataset with the URLs to access the data used in the work, and the annotations at the sentence level. In this way, the information available is fully sufficient to be able to replicate the same experiments presented in this article with the only effort of downloading the content of a set of URLs, using the wget command for example. It can be found here: https://doi.org/10.5281/zenodo.10802196.

Major points:

In the section on data collection (Section 4.1), the authors claim to have collected the articles from Snopes and Politifact by looking for those labelled with the category "medical", "health-check" and "coronavirus". However, the distribution of articles per category is not presented, raising the issue of whether the corpus is unbalanced and therefore not representative of the whole health domain. For example, the corpus may contain mostly documents related to COVID-19. Please provide the statistics in this section.

- Thank you for pointing this out. The categories mentioned in section "Data collection and selection of news items" are established by verification entities and are not always consistent with respect to the items they contain. In fact Snopes, which is our main source, has changed these categories several times. Instead, we have added Table 3, which shows the distribution of topics by news item, which better reflects the content of the dataset. As can be seen in the Table 3, there is quite a variety of topics.

For triple extraction, the authors state that a set of verbs was manually selected (lines 608-612) in order to parse the sentences. However, there is no justification on the criteria used for this selection. It would be explanatory for the reader to see a list of these verbs in the Appendix or at least to understand the

rationale behind the selection.

- Thank you for pointing this out. We have added a new appendix S3 that we reference from section "Fact-checking classification", where the verbs used appear. This appendix indicates that the objective of using them is to capture triplets related to diseases, causes, symptoms, treatments, and changes in vital signs. We have also added a clarification further down in the same section, explaining the reason for the use (with lower priority than the previous ones) of the verbs be and have:

"If we have not yet found predicate in the sentence, a third process looks for predicates with the verbal constructions modal+be (can be, should not, ...), be (is, are, won't have been, ...) or have (has, will, have, ...), extracting subject and object from the predicate found as described above. With this we seek to capture more generic relationships of the "is-a" or "has-a" type, which can also be present in any sentence."

One of the major issues is the fact that in Section 5.3, in Table 13, only results obtained by applying FC on sentences manually annotated as relevant are presented. This overshadows the fact that the pipeline should be applied by first automatically classifying the sentences as worthy (CW). In fact, as tackled in the Discussion section, the application of CW in tandem with FC can cause a cascade of errors which has an impact on the final results of fake news detection. As a consequence, it would be appropriate to put the results of the whole pipeline on KEANE in order to support the statements of this paper.

- We have retrained the models based on the annotation made by the best check-worthiness model. In this way, as reviewer mentions, errors accumulate from one stage to the next. The results are displayed in the new "Full pipeline classification results" section.

We can see in the included results tables that, in the test set our sentence-based model results (0.657) are very close to those obtained with manual check-worthy annotation (0.682). It is expected that these results will improve as the dataset is increased. On the other hand, on the ReCOVery dataset, which has a much larger number of instances to evaluate, our model is competitive with an average value of F1 (0.661) higher than that obtained by most transformer models.

The paper lacks a thorough error analysis to investigate the validity of the results obtained. This section should discuss for which reason sentences which contain facts are not considered worthy and cases of predicted false worthiness.

Moreover, it would be interesting to analyse the cases in which the FC model produced incorrect predictions in order to investigate which typology of sentences raise an issue for the model and mitigate the risk of bias.

- Thank you for pointing this out. We have added the new "Error analysis" section where we study the errors made by the classifiers from various perspectives.

As Figure 5 shows, many words can be mapped to wrong CUI terms. Tackling this issue in the discussion is necessary to give enough scientific validity to the algorithm used. Moreover, a way to evaluate the CUI mapping should be proposed as future work. mitigate the risk of bias.

- Thank you for pointing this out. It is true that Figure 5 shows some tokens, such as the predicate of the first sentence, that could have been mapped better. The CUI extraction work is performed by the external MetaMap application[1] which also contains the UMLS Metathesaurus. Since the extraction of these terms is not one of the objectives of our research, we cannot develop for this work something that improves MetaMap, but we have added as future work to study at least some mechanism for discarding tokens that semantically do not match. Despite these inconsistencies, we believe that CUIs provide new features that are useful in classification.

Minor points:

In the Introduction, the statistics used to justify the need for fake news detection in the health domain are based on an article published in 2013. It would make the motivation stronger to use more up-to-date data.

- Thank you for pointing this out. We have updated the first statistic in that paragraph with reference to a more recent publication. Unfortunately we have not been able to find more up-to-date information on the second statistic that talks about the percentage of self-diagnoses. Since we are interested in showing this information, we have maintained the 2013 reference for this second statistic:

"According to [8] ~~59% of adults~~ 58.5% of U.S. adults look for health information online and 35% use it to self-diagnose [3], avoiding going to a doctor."

[1] https://lhncbc.nlm.nih.gov/ii/tools/MetaMap.html

Line 58 starts with "Paper outline". However, this is probably a typo.

- Thank you for pointing this out. We have removed that sentence because it is redundant with the following one.

On line 350-351 and lines 369-371, the authors claim that the results on news detection are high enough to automatically annotate new articles; however, I advise to make this assertion less strong since the model does not achieve enough accuracy to operate autonomously.

- We have modified that sentence and a similar one to moderate the statement made about the classifier's performance:

*"Although this strategy is not capable of surpassing the results obtained with a transformer model fed with the first n tokens of the news (F1: 0.747), ~~they are high enough to be useful in the **evaluation of new articles**~~ it helps us to identify a large proportion of suspected fake articles (F1 = 0.703)."*

" ~~It's a high enough value to evaluate new articles which allows us to use it as the last stage of the pipeline~~ It is a value high enough to evaluate new articles, which allows us to use it as the last stage of the process, always considering that the result obtained is an estimate and should be subsequently verified by a human evaluator."

On Table 2, there is a typo on the final news item count. Why is it 327 and not 227? Does this mean that 100 articles in the corpus cannot be evaluated?

- Thank you for pointing this out. A 1 had been omitted. The fake news items are actually 181.

There is an inconsistency for the label FRC for multi-label CW. In Section 2.1, it is stated that a sentence is labelled as FRC if it contains more than a fact. Instead, on lines 493-494 it is written that sentences with facts too complex to be analysed are labelled as FRC. Making this definition consistent throughout the paper will improve its readability.

- Thank you for pointing this out. We have modified the sentence in the "Compiled dataset structure and statistics" section, clarifying what we mean by factual relevant and complex sentences:

"The final dataset consists of 10335 sentences divided into 1132 factual rel-

evant sentences whose fact-checking values has been annotated, and 9203 not factual, factual not relevant, not applicable, or factual relevant sentences that contain more than one claim and are therefore too complex to be analyzed."

Responses to the comments of reviewer 2

Reviewer #2: The manuscript's objectives are 3-fold:

1) propose a framework for collecting health-related articles for the tasks of check-worthiness and fact-checking;

2) generate a corpus from the collected articles;

3) design a neural model to classify the articles.

The framework uses a classic text classification pipeline, offering overall very little novelty.

There are a lot of works published that propose how to use word embeddings [1], document embeddings [2], transformers [3], and sentence transformers [4] for the task of misinformation detection. Also, the current literature discusses how network information can improve the detection task [5]. How is this work compared with the proposed model?

- In the "Related work" section we review those different approaches that use different strategies and technologies applied to the problem of Fake News. Our way of approaching this problem is to analyze only the content without the need of resorting to network information. The last paragraph of the "Fake news detection challenges" section is where we compare our approach with the alternatives described previously:

"The system that we propose in this paper can be considered a claim based system which also uses latent features, although differs from the previous ones in several aspects: 1) we carry the burden of detecting false news at the sentence level but we maintain as objective the evaluation of the news item, 2) we in turn break down sentences into subject, predicate and object by exploring how transformer models can use this information to evaluate the truthfulness of sentences, 3) we enrich the information of these sentences by means of terms proper to the domain of health (UMLS), and 4) we propose an ensemble model that allows integrating the results of the two previous points."

Also, why any of the following were not used: word embeddings, document

embeddings, or sentence transformers?

- Using embedding layers or sentence transformers to encode linguistic features of words or sentences is certainly a possibility if a classification method based on LSTMs, CNNs, etc., is implemented behind it. In our case we have not considered this option since we use transformer models for classification and these already internally incorporate an embedding layer that does this encoding work. We could have used something like this in the FFNN components of the ensemble but we have preferred to use simpler TF-IDF vectors in the hope that they provide complementary information to that provided by the transformer components. In short, we do not use word embeddings, document embeddings, or sentence transformers extrinsically but we do use embeddings and transformers as part of our approach.

After detecting that a piece of information is fake, what should we do with this information? Leave it as it is? There is a large body of work that discusses how we can mitigate the spread of fake news using network immunization, such as proactive approaches [6], tree-based approaches [7], community-based approaches [8], or real-time approaches [9]. I consider this to be very important both in the discussion section and for future work.

- Thank you for your suggestion of future work. Although our proposal is based on content-focused detection of fake news, mitigation and immunization strategies are very interesting lines of research that are connected as subsequent steps in the process.

We have modified the "Related Work" section to mention them by adding references to some works on immunization and misinformation mitigation. To maintain consistency with the rest of the section, which deals with detection only, these references have been added to the opening paragraph:

"In this section we review previous works related to the detection of fake news. There are other lines of research mainly based on the analysis of the spread of disinformation on social networks, which go beyond detection and propose mitigation strategies for the effects of this disinformation [7][5] or the immunization of the nodes causing it [1][4]. In this work, however, we focus on detection. We divide ~~them~~ detection approaches into two large groups: those that contemplate the article as a whole, and those that analyze their claims to determine the truth value of them. We also show different examples of datasets oriented to this task

that have appeared over time."

The annotation process seems mostly automatic, only at the end does a user check it.

For the Check-worthy annotation, I find it superficial to annotate only 100 sentences.

- Annotations are automatic for new articles although they require confirmation, but all sentences from the training and test datasets used in this paper have been annotated manually. This means that in the 10,335 sentences the check-worthiness annotation has been carried out manually, and of these, in the 1,132 that are factually relevant, the fact-checking annotation has also been carried out manually.

For the Fact-checking annotation, it is not clearly explained how the manual annotation was done.

- We have modified Section "Fact-checking annotation" hoping to make it clearer how to carry out fact-checking annotation of sentences:

"At this essential stage in the process of determining the veracity of a news item, only sentences that have been previously annotated as factual and relevant arrive.

As in the previous stage, a manual annotation of the approximately one thousand sentences of this type has been made. ~~, in this case based on the overall assessment and the justifications provided by the website that reviewed the news item. In this process we have considered what is the claim or claims according to the fact-checking website that make the article false and also what other sentences can be true even if the article as a whole is false.~~ To do this, in each news item we have reviewed the evaluation of the article made by the verification entity. For example, Snopes usually identifies suspicious claims within the article and their level of truthfulness. Based on this, we have searched for the sentences where these claims appear to label them as false. We have considered the rest of the sentences true. In specific cases where false claims were not clearly identified, we have turned to reliable sources such as Wikipedia or MedlinePlus to verify suspicious sentences."

For the CheckThat! 2022 results (table 6), the individual papers should also be

cited in the table, not only the paper that presents the overview of the challenge.

- Thank you for pointing this out. Citations have been added to all the teams that appear in Tables 5 and 7 and presented a paper.

The results do not seem very promising. I find the scores very low compared with the current literature. Why is this happening?

- The results of the models depend on the difficulty offered by the dataset on which they are evaluated. In a CheckThat! shared task [6] that is quite similar to the validation approach used in this paper, the participants with the best results obtained F1 measures lower than 0.34 in multiclass fake news detection. In tests carried out on our dataset also with fake news multiclass classification we obtained F1 values higher than 0.5.

The experiments seem shallow:
1. There is no hyperparameter tuning evaluation for the models employed. The authors only use the "most promising" hyperparameters

- We have used the expression "most promising" since, as we explain in the document, we first do a grid search for the transformer and FFNN models with different hyperparameters and random seeds, and then we load those trained models into the ensemble to perform a second grid search. The fact that they are the best hyperparameters for these models alone does not have to imply that they are the best as a component of the ensemble. Unfortunately, checking all possible combinations of hyperparameters of the components and the ensemble itself is infeasible. Anyway, we have added some additional detail about the hyperparameters used in that paragraph:

"For the ensemble model we have set the most promising hyperparameters of the previous two classifiers, transformer and FFNN. From the transformer we have selected the BERT Cased, ALBERT, BioBERT and BlueBERT pretrained models, no vocabulary expansion, and a maximum of 10 epochs. The first four pretrained models have been evaluated in the first transformer component of the ensemble and BlueBERT has been configured in the second transformer component that accepts SPO triplets formed by the descriptions of the CUIs. And from the FFNN we have used the tanh activation function, a hidden layer size of 100, without class weights compensation, and 250 epochs at most. In the three components of the ensemble, the random seed that had a higher F1 value was used. Based on this configuration, we have evaluated the different input types and hy-

perparameters: pretrained model for the first transformer component of the ensemble, one or two hidden layers, dropout of 0, 0.2 and 0.5, and a maximum of 10 epochs."

2. Are the results obtained using cross-validation?

- No cross validation is used. As indicated in the "Check-worthy classification" section, the dataset has been randomly partitioned into sets: *training+dev* and *validation*. That same partition, as mentioned in the sections "Sentence fact-checking classification results" and "Fake news classification results" has been maintained for the fact checking classification of sentences and fake news classification of full articles. The reason for doing this is because we need that if an item belongs to the set like *training*, its sentences also belong to the same data set. If we were to randomly sample sentences for cross validation, this would not be true and an article could have sentences scattered across the *training* and *test* sets. If we used these sentences to determine the veracity of the article, some of them would belong to the *training* set, which would distort the results by using instances "observed" during training. Furthermore, in shared tasks like CheckThat! it is common to have the training and test sets fixed.

How many training iterations were used?

- The number of epochs is variable as mentioned in the "Check-worthy classification" section. In section "Sentence check-worthy classification results" it is indicated that there is an early stopping mechanism. We have modified that paragraph by adding that the maximum number of epochs is 10:

"An early stopping mechanism has been implemented that stops training if the F1 measure has not been improved in the dev dataset in the last 3 epochs, recovering the saved model that had the best result for that configuration. The maximum number of epochs has been set to 10."

In the "Sentence fact-checking classification results" section the maximum number of epochs for each model used was already indicated (250 for FFNN and 10 for transformers).

What are the mean and the standard deviation obtained for each evaluation metric on the test set?

- The standard deviation has been added for each of the measurements shown in the different tables. This is indicated in the "Sentence check-worthy classifica-

tion results" section, which is the first mention in the paper of this calculation on ten random seeds:

"Furthermore, in order to alleviate the performance variability inherent to the transformer models [2], each model has been executed for ten different random seeds and the average of the accuracy, precision, recall and F1 measures has been calculated to elaborate the results shown in Table 8 (binary classification) and Table 9 (multi-class classification). Next to each measurement, the standard deviation obtained on these ten random seeds is also shown."

3. There is no time performance evaluation.

- In the section "Pipeline training and inference times" we indicate both the training times of the different models that make up the ensemble and of the ensemble itself. The time it takes to evaluate a new article through the pipeline is also indicated:

In the check-worthy task, training a transformer model such as BERT Base Cased has required almost 7 minutes in binary classification (6 epochs), and just over 16 minutes in multiclass classification (10 epochs). In the sentence fact-checking task we have verified that an FFNN model can be trained in less than 10 seconds (8 epochs), a BERT-type transformer uses a little more than a minute (6 epochs), and the training of the ensemble model is performed in just under a minute (5 epochs). Therefore, training an complete ensemble model with two transformers and one FFNN does not take more than 4 minutes. In the fake news detection task, the time needed to assess the veracity of an entire article based on its sentences is only a few seconds.

Regarding the inference times, the processing of a complete article with 96 sentences of which 5 are factually relevant needs a little over 12 minutes. A significant part of this time is consumed by the MetaMap process that extracts medical identifiers from the text of sentences.

4. Ablation testing is missing.

- They are not mentioned explicitly but in fact, we are already performing an ablation test. Our most complete fact checking model with all the features is the ensemble composed of two transformers and one FFNN (*Ens. FFNN-Tr1-Tr2*). We have also evaluated the ensemble without the FFNN (*Ens. Tr1-Tr2*). And finally we have separately evaluated the FFNN and the different transformer

models.

I highly recommend that the GitHub repository be populated for reproducibility purposes.

- We have published the source code of the framework in the following repositories: https://github.com/jrmtnez/hnfc-agent and https://github.com/jrmtnez/hnfc-site.

Also, the code is useless without the collected dataset. Please offer a publicly available repository with the proposed dataset.

- Given the nature of the data used in this work, which may be subject to distribution restrictions, we have published a dataset with the URLs that allow access to the original news items, and for each of them, the sentence-level annotation of both: fact-checking and check-worthiness. In this way, the information available is fully sufficient to be able to replicate the same experiments presented in this article with the only effort of downloading the content of a set of URLs, using the wget command for example. It is available at the following URL: https://doi.org/10.5281/zenodo.10802196.

Please do a thorough spell-checking of the article before resubmitting.

- Thank you for pointing this out. We have revised the document, correcting some errors and simplifying expressions to make it more concise.

Bibliography

[1] Ozgur Coban, Ciprian-Octavian Truică, and Elena-Simona Apostol. CONTAIN: A Community-based Algorithm for Network Immunization, March 2023. arXiv:2303.01934 [cs].

[2] Jesse Dodge, Gabriel Ilharco, Roy Schwartz, Ali Farhadi, Hannaneh Hajishirzi, and Noah Smith. Fine-Tuning Pretrained Language Models: Weight Initializations, Data Orders, and Early Stopping. *arXiv:2002.06305 [cs]*, February 2020.

[3] Susannah Fox and Maeve Duggan. Health Online 2013, January 2013.

[4] Ai-Wen Li, Xiao-Ke Xu, and Ying Fan. Immunization strategies for false information spreading on signed social networks. *Chaos, Solitons & Fractals*, 162:112489, September 2022.

[5] Akrati Saxena, Cristina Gutiérrez Bierbooms, and Mykola Pechenizkiy. Fairness-aware fake news mitigation using counter information propagation. *Applied Intelligence*, 53(22):27483–27504, November 2023.

[6] Gautam Kishore Shahi, Julia Maria Struß, and Thomas Mandl. Overview of the CLEF-2021 CheckThat! Lab: Task 3 on Fake News Detection. page 18, 2021.

[7] Ciprian-Octavian Truică, Elena-Simona Apostol, Radu-Cătălin Nicolescu, and Panagiotis Karras. MCWDST: a Minimum-Cost Weighted Directed Spanning Tree Algorithm for Real-Time Fake News Mitigation in Social Media. *IEEE Access*, 11:125861–125873, 2023. arXiv:2302.12190 [cs].

[8] Xun Wang and Robin A. Cohen. Health Information Technology Use Among Adults: United States, July-December 2022. Technical report, National Center for Health Statistics (U.S.), Hyattsville, MD, October 2023.
