# Supplementary material for: Building a framework for fake news detection in the health domain
Source: PLoS One. 2024 Jul 8;19(7):e0305362. doi: 10.1371/journal.pone.0305362 (PMC11230534; doi:10.1371/journal.pone.0305362)
Supplement: Attachment — Submitted filename: response_to_reviewers_R2.pdf [file pone.0305362.s004.pdf]

Authors’ Response to the Reviewer’s Comments

Article:: PONE-D-23-40818R1
Title: Building a Framework for Fake News Detection in the Health Domain
Authors: Juan R. Martinez-Rico, J. Martinez-Romo, and Lourdes Araujo

The authors thank the editor and reviewers for their comments which greatly helped them to improve the contents of the paper. We have prepared a revised version of the paper according to their suggestions. Changes and new parts appear in red in this version.

Next, we will show the comments/suggestions from the reviewer in italics and our response in regular typeface.

Responses to the comments of the editor

Thank you so much for your comments. We have followed the suggestions and indications of the editor and reviewers and have modified the article, mainly in the following aspects:

1. I have just received the second reviewer’s revisions, which I fully agree with regarding the need to cite some work on word embedding, transformers, and document embedding to detect false information, so that your paper takes this relevant literature into account.

Thank you so much for your comments. We have followed the suggestions and indications of the editor and reviewers and have modified the article:

- We have expanded the “Related work” section by including references to various works on embeddings and Transformers applied to disinformation detection. The modifications made are detailed in the response to the second reviewer.

- We have also reviewed the list of references verifying that none have been retracted.

Responses to the comments of reviewer 1

The authors have responded with sufficient clarity to all the critical issues in the manuscript and I see no impediment to the publication of the article as it is

now presented.

-Thank you so much for your comments and feedback. We really appreciate it.

Responses to the comments of reviewer 2

Although the authors answered most of my comments, I still think that the manuscript is missing a large chunk of literature regarding word embeddings [1], transformers [2], and document embeddings [4] for detecting fake information. I recommend that the authors mention some of these research endeavors in their related work section so the study is complete, otherwise, it would seem that a large chunk of work was ignored.

[1] https://scholar.google.com/scholar?q=word+embeddings+misinformation+detection
[2] https://scholar.google.com/scholar?q=transformers+misinformation
[3] https://scholar.google.com/scholar?q=fake+news+document+embeddings

- Thank you very much for your comments and suggestions. Certainly including these references completes the vision of the different works that have been carried out in this area of research. We have expanded the “Related Work” section within the subsection dedicated to content-based methods, including references to embeddings applied to disinformation detection. We have also added references to works that have used Transformers for this task, either as classifiers or to generate contextual information. The changes made to this subsection and Table 1 are shown in red below:

Document content based: *The first strategy considers only the textual content of the article by trying to associate misleading content with certain writing styles [1], using features such as bag-of-words vectors, part-of-speech tags, or Probabilistic Context Free Grammars to carry out this task. Text analysis techniques such as LIWC [2], and discourse level features that analyze the differences in terms of coherence and structure between deceptive and truthful narratives [3] have ~~also~~ been used as features to detect misleading content. ~~We can even include in this section the Transformer models~~ [4]~~, that allow us to obtain state-of-the-art results with hardly any feature design.~~ Methods such as word embeddings [6] have also been studied, which, when extended to the entire document, provide their ability to analyze the context in which a word appears, in order to identify typical patterns*

of disinformation and sensationalist language. It has even been found that the correct generation of these embeddings by training with the appropriate data [5], allows obtaining with simple classification models, results similar to or superior to those obtained with more complex models [7]. Another strategy used to detect disinformation based on the content of the document is the currently ubiquitous Transformer models [8]. On the one hand, taking advantage of its capabilities to extract latent features from the text without the need for elaborate feature design, and apply using transfer leaning the information obtained during its pre-training together with a fine adjustment in the target datasets to this detection task [4] [9]. On the other hand, Transformers can also be used to generate contextual embeddings that capture the meaning of words, as well as subtle cues that can characterize misleading information. In these cases, transformers are typically used as components of an ensemble model [10] that contains other components dedicated to the processing of intermediate information and the final classification of documents. These methods can provide good results although ~~the former~~ those mentioned at the beginning require a careful design of features, and the results of the Transformer models can be very difficult to explain. However, the main disadvantage of all these methods is that they could be circumvented by an agent who can mimic the style of legitimate news even by incorporating misleading content. It is also worth mentioning in this group the existence of multimodal approaches that include other types of resources such as the images contained in the article [11] [12].

Table 1: **Fake news detection approaches.**

**Method**	**Ref.**	**Document**		**Claim**	
		Content	Context	Structured	Unstr.
Writting styles	[1]	✓			
Text analysis	[2]	✓			
Discourse analysis	[3]	✓			
Latent features (Transformers)	[4]	✓			
Embeddings	[5]	✓			
Propagation	[13]		✓		
Publishers, news, users relations	[14]		✓		
User features	[15]		✓		
News-topics inconsistency graphs	[16]	✓	✓		
Content + social events integration	[17]	✓	✓		
Triplet search in KGs	[18]			✓	
Rules extracted from fact patterns	[19]			✓	
Similarity with search engines results	[20]				✓
Claim-evidence embeddings	[21]				✓

Bibliography

[1] Feng S, Banerjee R, Choi Y. Syntactic stylometry for deception detection. In: Proceedings of the 50th Annual Meeting of the Association for Computational Linguistics (Volume 2: Short Papers); 2012. p. 171–175.

[2] Mihalcea R, Strapparava C. The lie detector: Explorations in the automatic recognition of deceptive language. In: Proceedings of the ACL-IJCNLP 2009 Conference Short Papers. Association for Computational Linguistics; 2009. p. 309–312.

[3] Rubin VL, Lukoianova T. Truth and deception at the rhetorical structure level. Journal of the Association for Information Science and Technology. 2015;66(5):905–917.

[4] Jwa H, Oh D, Park K, Kang JM, Lim H. exBAKE: Automatic Fake News Detection Model Based on Bidirectional Encoder Representations from Transformers (BERT). Applied Sciences. 2019;9(19):4062. doi:10.3390/app9194062.

[5] Ilie VI, Truica CO, Apostol ES, Paschke A. Context-Aware Misinformation Detection: A Benchmark of Deep Learning Architectures Using Word Embeddings. IEEE Access. 2021;9:162122–162146. doi:10.1109/ACCESS.2021.3132502.

[6] Mikolov T, Yih Wt, Zweig G. Linguistic regularities in continuous space word representations. In: Proceedings of the 2013 conference of the north american chapter of the association for computational linguistics: Human language technologies; 2013. p. 746–751. Available from: `https://aclanthology.org/N13-1090.pdf`.

[7] Truică CO, Apostol ES. It's all in the embedding! fake news detection using document embeddings. Mathematics. 2023;11(3):508.

[8] Vaswani A, Shazeer N, Parmar N, Uszkoreit J, Jones L, Gomez AN, et al. Attention is all you need. In: Advances in neural information processing systems; 2017. p. 5998–6008.

[9] Aggarwal A, Chauhan A, Kumar D, Verma S, Mittal M. Classification of fake news by fine-tuning deep bidirectional transformers based language model. EAI Endorsed Transactions on Scalable Information Systems. 2020;7(27):e10–e10.

[10] Truică CO, Apostol ES. Misrobærta: transformers versus misinformation. Mathematics. 2022;10(4):569.

[11] Cui L, Wang S, Lee D. SAME: sentiment-aware multi-modal embedding for detecting fake news. In: Proceedings of the 2019 IEEE/ACM International Conference on Advances in Social Networks Analysis and Mining. Vancouver British Columbia Canada: ACM; 2019. p. 41–48. Available from: https://dl.acm.org/doi/10.1145/3341161.3342894.

[12] Alam F, Cresci S, Chakraborty T, Silvestri F, Dimitrov D, Martino GDS, et al. A Survey on Multimodal Disinformation Detection. In: Proceedings of the 29th International Conference on Computational Linguistics. Gyeongju, Republic of Korea: International Committee on Computational Linguistics; 2022. p. 6625–6643. Available from: https://aclanthology.org/2022.coling-1.576.

[13] Davoudi M, Moosavi MR, Sadreddini MH. DSS: A hybrid deep model for fake news detection using propagation tree and stance network. Expert Systems with Applications. 2022;198:116635. doi:10.1016/j.eswa.2022.116635.

[14] Shu K, Wang S, Liu H. Beyond news contents: The role of social context for fake news detection. In: Proceedings of the twelfth ACM international conference on web search and data mining; 2019. p. 312–320.

[15] Shu K, Zhou X, Wang S, Zafarani R, Liu H. The role of user profiles for fake news detection. In: Proceedings of the 2019 IEEE/ACM international conference on advances in social networks analysis and mining; 2019. p. 436–439.

[16] Xu J, Zadorozhny V, Zhang D, Grant J. FaNDS: Fake News Detection System using energy flow. Data & Knowledge Engineering. 2022;139:101985. doi:10.1016/j.datak.2022.101985.

[17] Raza S, Ding C. Fake news detection based on news content and social contexts: a transformer-based approach. International Journal of Data Science and Analytics. 2022;13(4):335–362. doi:10.1007/s41060-021-00302-z.

[18] Ciampaglia GL, Shiralkar P, Rocha LM, Bollen J, Menczer F, Flammini A. Computational Fact Checking from Knowledge Networks. PLOS ONE. 2015;10(6):e0128193. doi:10.1371/journal.pone.0128193.

[19] Lin P, Song Q, Shen J, Wu Y. Discovering Graph Patterns for Fact Checking in Knowledge Graphs. In: Pei J, Manolopoulos Y, Sadiq S, Li J, editors. Database Systems for Advanced Applications. vol. 10827. Cham: Springer International Publishing; 2018. p. 783–801. Available from: http://link.springer.com/10.1007/978-3-319-91452-7_50.

[20] Karadzhov G, Nakov P, Màrquez L, Barrón-Cedeño A, Koychev I. Fully Automated Fact Checking Using External Sources. In: Proceedings of the International Conference Recent Advances in Natural Language Processing, RANLP 2017; 2017. p. 344–353.

[21] Xu W, Wu J, Liu Q, Wu S, Wang L. Evidence-aware Fake News Detection with Graph Neural Networks. In: Proceedings of the ACM Web Conference 2022. WWW '22. New York, NY, USA: Association for Computing Machinery; 2022. p. 2501–2510. Available from: https://doi.org/10.1145/3485447.3512122.
